# Effectiveness of Dose Adjustment of Insulin in Type 2 Diabetes among Hemodialysis Patients with End-Stage Renal Disease: A Randomized Crossover Study

**DOI:** 10.1155/2019/6923543

**Published:** 2019-11-13

**Authors:** Amol Singhsakul, Ouppatham Supasyndh, Bancha Satirapoj

**Affiliations:** Division of Nephrology, Department of Medicine, Phramongkutklao Hospital and College of Medicine, Bangkok 10400, Thailand

## Abstract

Determining insulin requirements for hemodialysis patients with end-stage renal disease (ESRD) is difficult. We performed a randomized crossover study among type 2 diabetes (T2DM) patients with ESRD on continuous hemodialysis and receiving standard insulin for glycemic control. The patients were randomized in 2 groups: daily insulin needed on the day after hemodialysis and a 25% decrease in daily insulin needed on the day after hemodialysis. A total of 51 T2DM patients with ESRD were enrolled. The adjusted-insulin group had higher plasma glucose levels at the 2nd hour of dialysis than those of the nonadjusted-insulin group. Incidence of hypoglycemia per dialysis session (3.3% vs. 0.7%, *P* = 0.02) and symptoms related to hypoglycemia (6.9% vs. 0.7%, *P* = 0.001) were more frequent in the nonadjusted-insulin group. A reduced insulin administration of 25% among T2DM patients undergoing hemodialysis on the day of dialysis was associated with sustained glycemic efficacy and the production of fewer hypoglycemic symptoms. This trial is registered with TCTR20180724002.

## 1. Background

Type 2 diabetes mellitus (T2DM) is the leading cause of end-stage renal disease (ESRD) and dialysis therapy [1, 2]. The prevalence of nephropathy in T2DM is still rising dramatically, with concomitant increases in associated mortality and cardiovascular complications [3]. Patients with ESRD from T2DM challenge nephrologists because they have the greatest number of comorbid conditions and the greatest dependency in activities of daily living. Glycemic therapy among patients with diabetes has been shown to improve outcomes, especially microvascular complications among patients without advanced kidney disease [4, 5]. The benefit of tight glucose control on renal progression in advanced kidney disease is less well studied, and hypoglycemia is common in this population [6]. Therefore, data are scarce on how glycemic control should best be treated in T2DM patients with ESRD.

Approximately 30 to 80% of systemic insulin is metabolized particularly in the kidney [7]. Renal insulin clearance decreases as glomerular filtration rate decreases to less than 15 to 20 mL/min/1.73 m^2^ [8]. Hepatic clearance of insulin is also decreased in patients with uremia. Deficient gluconeogenesis, along with malnutrition, deficient catecholamine release, and impaired renal insulin degradation and clearance, can contribute to decreasing insulin requirements and frequent hypoglycemia among patients with ESRD [9, 10]. In contrast, hemodialysis improves insulin sensitivity and also insulin clearance, making it more difficult to determine insulin requirements for patients with ESRD who are undergoing maintenance hemodialysis [11]. Whether dialysis has a potential effect on pre- to postdialysis day changes in exogenous insulin requirements remains uncertain.

Initial data has demonstrated a significant 25% reduction in basal insulin requirements the day after dialysis compared with the day before using 24-hour euglycemic clamp monitoring [12]. Currently, there are evidence-based recommendations available to adjust insulin dose on dialysis day among T2DM patients. This study was undertaken to demonstrate the effectiveness of reduced insulin dosage on the day of dialysis for T2DM patients with ESRD who were undergoing maintenance hemodialysis.

## 2. Patients and Methods

The study employed a four-week randomized, controlled, crossover design conducted among T2DM patients with ESRD undergoing continuous hemodialysis and receiving standard insulin for glycemic control at Phramongkutklao Hospital, Bangkok, Thailand. The study was approved by the Institutional Review Boards of the Phramongkutklao Hospital and the College of Medicine. Using block randomization, patients were assigned to one of two double-blinded treatment groups. A computer-generated randomization procedure, in blocks of four, was used. Inclusion criteria comprised being 18 years or older, diagnosis of T2DM, on hemodialysis three sessions weekly and for at least three months, stable insulin dosage once daily for at least three months, and stable glycemic treatment with HbA1c < 8% for at least three months. Exclusion criteria included diagnosis of type 1 diabetes, pregnancy, active malignancy, being physically challenged, active or chronic infection within three months of starting the study, and symptomatic hypo- or hyperglycemia within three months. All subjects gave their informed consent before enrolling.

**Figure 1:**
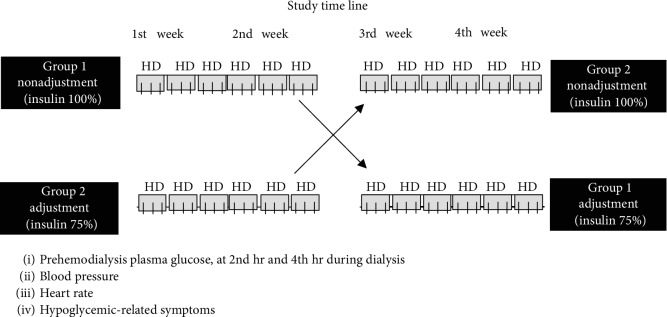
Study flow chart.

Eligible patients were randomly assigned to two groups. One group consisted of 26 patients treated with a standard dose of insulin as a patient prescription, while the other group consisted of 25 patients treated with an adjusted dose of insulin (25% decrease of prescription). All patients received nutritional advice about carbohydrate intake in a standardized three-meal day. Both groups of patients continued regular hemodialysis, three sessions weekly. During each session, patients had blood samples taken prehemodialysis and during hemodialysis on the second and third hours. After two weeks of study, patients in the standard-dose insulin group were switched to the adjusted-dose insulin group and patients on the adjusted-dose insulin group were switched to the standard dose. Regular hemodialysis was then continued and blood samples taken as with the first two weeks of study. During every session of hemodialysis, patient blood pressure, heart rate, abnormal symptoms, and any instance of hospitalization were recorded as shown in Figure 1.

### 2.1. Biochemical Measurements

All subjects underwent laboratory blood tests at baseline including lipid profiles, liver function, complete blood count, blood urea nitrogen, creatinine, and electrolytes, and then *KT*/*V* was calculated. Plasma glucose levels were measured at prehemodialysis and during hemodialysis on the second and third hours. Hypoglycemia was defined as plasma glucose level < 70 mg/dL.

### 2.2. Safety Monitoring

Adverse events that were or were not considered related to insulin treatment were monitored during hemodialysis by the nursing staff at the dialysis center. The patients were systematically questioned during each session concerning their experiences of adverse events during dialysis. Patients also underwent blood drawing to confirm whether the symptoms were related or nonrelated to abnormal blood glucose levels.

### 2.3. Statistical Analysis

Statistical analyses were performed using SPSS version 15.0. Descriptive statistics were used to summarize demographics and baseline characteristics. The comparability of treatment groups was assessed using a two-way analysis of variance. Data were presented as mean ± SD and mean changes. Chi-square tests, independent *t*-tests, and paired *t*-tests were used to compare the plasma glucose and clinical parameters between two groups and within group. All results were considered significant when *P* was <0.05.

## 3. Results

A total of 51 patients with ESRD on regular hemodialysis three times weekly, mean age of 61.2 ± 10.6 years, and a mean dialysis vintage of 4.0 ± 2.3 years were enrolled. Mean prehemodialysis plasma glucose level was 171.3 ± 40.3 mg/dL, and the mean hemoglobin level A1C was 7.0 ± 0.8% (Table 1). On average, the total doses of insulin in the nonadjusted-insulin group and the adjusted-insulin group were 15.1 ± 8.2 and 12.4 ± 7.8 U/day (*P* = 0.073).

### 3.1. Changes of Plasma Glucose Level

At baseline before treatment, no significant differences of plasma glucose were observed in both groups. Over four hours of treatment, the mean plasma glucose level decreased significantly in both groups (*P* < 0.05). Over two hours of treatment, the adjusted-insulin group had a higher plasma glucose level at the 2nd hour of dialysis than the nonadjusted-insulin group (154.2 ± 37.5 vs. 136.9 ± 14.1, *P* = 0.035). However, the plasma glucose level at the 4th hour of dialysis was comparable in both groups and the percentage of reduced plasma glucose > 30% over four hours of treatment was similar in both groups (Table 2).

### 3.2. Hypoglycemic-Related Symptoms during Dialysis

**Figure 2:**
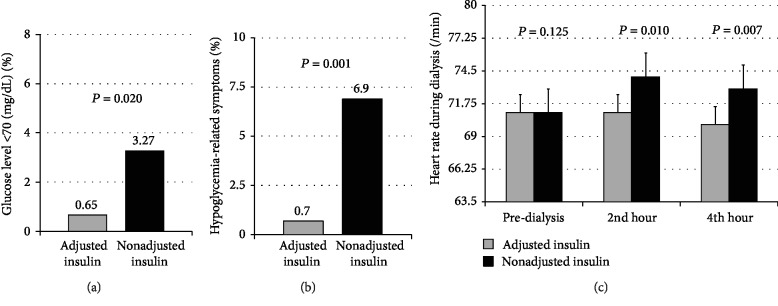
Hypoglycemia-related symptoms during hemodialysis. (a) The incidence of hypoglycemia per dialysis session was higher in the nonadjusted-insulin group than in the adjusted-insulin group (10/306 (3.3%) vs. 2/306 (0.7%), *P* = 0.020). (b) Symptoms related to hypoglycemia were more frequent in the nonadjusted-insulin group than in the adjusted-insulin group (21/306 (6.9%) vs. 2/306 (0.7%), *P* = 0.001). (c) The nonadjusted-insulin group produced a significant increase in heart rate during the 2nd hour of dialysis compared with the adjusted-insulin group (*P* = 0.010).

Incidence of hypoglycemia per dialysis session (3.3% vs. 0.7%, *P* = 0.02) and symptoms related to hypoglycemia (6.9% vs. 0.7%, *P* < 0.001) were more frequent in the nonadjusted-insulin group than in the adjusted-insulin group. Additionally, the nonadjusted-insulin group also produced a significant increase in pulse rate during the 2nd hour of dialysis, compared with the adjusted-insulin group (*P* < 0.01) (Figure 2). No differences of mean arterial blood pressure in both groups were found. Major adverse events including acute coronary syndrome and cerebrovascular disease were not reported in any group.

## 4. Discussion

The present study constitutes the first randomized, controlled, crossover study demonstrating the effectiveness of insulin reduction at 75% on the day of dialysis for glycemic control during hemodialysis among T2DM patients with ESRD. The main benefit was less incidence of hypoglycemia in the reduced insulin treatment group.

ESRD on dialysis exerts opposing forces on insulin secretion, action, and metabolism, often creating unpredictable serum glucose values [13]. Some patients presenting insulin resistance need more supplemental insulin [14]. In contrast, reduced renal gluconeogenesis and insulin clearance seen in ESRD may need lower doses of insulin treatment after hemodialysis [15]. Together, all these factors contribute to wide fluctuations in plasma glucose levels and increase the risk of both hyperglycemic and hypoglycemic events [13, 14, 16]. Clinical practice recommendations do not address the adjustment of insulin treatment among patients with ESRD undergoing hemodialysis [17]. Although greater insulin clearance on the day of dialysis may also influence the effectiveness of insulin and dialysis therapy improves uremia-associated insulin resistance [18], related studies, evaluating day-to-day variations of insulin needs among T2DM patients with ESRD receiving maintenance hemodialysis, showed a significant reduction of 25% in basal insulin requirements the day after dialysis compared with the day before [12]. Moreover, plasma glucose levels decreased significantly during hemodialysis [19–21]. In our study, data also showed a significantly lower plasma glucose level at the 2nd hour of the hemodialysis session and more hypoglycemic-related symptoms in the adjusted-insulin group, consistent with the data concerning improvement of insulin sensitivity after dialysis.

Approximately one-third of T2DM patients receiving insulin treatment did not need insulin after initiating hemodialysis at one year [22]. In addition, the frequency of hypoglycemic events during dialysis was higher than during the predialysis period [23]. This was consistent with our study in that the occurrence of hypoglycemic-related symptoms including plasma glucose < 70 mg/dL, palpitation, dizziness, and increased heart rate were significantly observed among patients in the nonadjusted-insulin group during hemodialysis. An increased heart rate was detected significantly among patients in the nonadjusted-insulin group during hemodialysis, especially in the 2nd and 4th hours of the session. Although it did not reach the tachycardia definition, almost all patients complained about palpitation. The symptom might have resulted from sympathetic surges and insulin effects with low glucose. Cholinergic and sympathetic presentations are early hypoglycemic responses when plasma glucose concentrations decrease among patients with poorly controlled glycemic control [24].

Insulin treatment among T2DM patients with ESRD is difficult to establish given the lack of pharmacokinetic studies concerning the various types of insulins among patients at different stages of CKD and the limited evidence for adjusting insulin [25]. Some authors recommend avoiding intermediate and long-acting insulins in ESRD [15]. However, our study revealed that intermediate-acting insulins showed fewer frequent hypoglycemic attacks (3.3%) during dialysis and fewer hypoglycemic symptoms after adjusting the dose of intermediate-acting insulins. Moreover, no serious hypoglycemic events occurred requiring medical intervention in our study.

Several limitations were found in this study. First, data on clinical and plasma glucose during 24 hours on the dialysis and nondialysis days were not monitored in the study. Second, the low dose of insulin required (<20 U/day) in both groups was likely to explain the lack of significant differences in the total dose of insulin in the adjusted and nonadjusted-insulin groups. Finally, two types of intermediate-acting insulin were used in the study, so it might be difficult to explain the overall outcomes of the study. Larger studies on populations undergoing hemodialysis will be required to determine whether our outcomes should be used for insulin adjustment on dialysis days to optimize glycemic control and avoid hypoglycemia.

## 5. Conclusion

In conclusion, our study demonstrated that reduced insulin administration by 25% on the day of dialysis among T2DM patients undergoing hemodialysis resulted in sustained glycemic control and stable plasma glucose levels with fewer hypoglycemic-related symptoms. The data suggested that the reduced insulin requirement among patients undergoing hemodialysis seemed to be related to improved insulin sensitivity and insulin clearance during hemodialysis.

## Figures and Tables

**Table 1:** Baseline characteristics of patients.

	*N* = 51
Female (*N*, %)	37 (72.6)
Age (years)	61.2 ± 10.6
Mean arterial blood pressure (mmHg)	80.2 ± 15.2
Heart rate (/min)	71.2 ± 11.2
Comorbid diseases (*N*, %)	
(i) Hypertension	48 (94.1)
(ii) Cardiovascular diseases	11(21.5)
Dialysis vintage (years)	4.0 ± 2.3
BUN (mg/dL)	64.2 ± 10.9
Serum creatinine (mg/dL)	7.8 ± 1.6
Serum albumin (g/dL)	3.9 ± 0.4
LDL-cholesterol (mg/dL)	107.2 ± 33.8
Single-pool *KT*/*V*	1.8 ± 0.3
Plasma glucose (mg/dL)	171.3 ± 40.3
Hemoglobin A1C (%)	7.0 ± 0.8%
Plasma sodium (mEq/L)	137.6 ± 3.3
Plasma potassium (mEq/L)	4.4 ± 0.8
Plasma chloride (mEq/L)	101.7 ± 2.9
Plasma bicarbonate (mEq/L)	23.9 ± 1.5
Insulin types	
(i) Humulin premix	43 (84.3)
(ii) Humulin N	8 (15.7)
Antihypertensive agents (*N*, %)	
(i) Calcium channel blockers	22 (43.1)
(ii) Beta blockers	18 (35.3)
(iii) Diuretics	19 (37.3)
(iv) Alpha blockers	6 (11.8)
(v) Renin angiotensin aldosterone blockers	9 (17.7)
(vi) Others	26 (50.9)

Data is presented as mean with SD and percentage.

**Table 2:** Changes of plasma glucose during dialysis treatment.

	Nonadjusted insulin (total dialysis treatment =306)	Adjusted insulin (total dialysis treatment =306)	*P* value
Insulin requirement (units/day)	15.1 ± 8.2	12.4 ± 7.8	0.073
Predialysis plasma glucose (mg/dL)	161.4 ± 38.8	173.4 ± 20.3	0.171
Plasma glucose at 2nd hour (mg/dL)	136.9 ± 14.1^∗^	154.2 ± 37.5^∗^	0.035
Plasma glucose at 4th hour (mg/dL)	121.9 ± 11.1^∗^	127.9 ± 20.4^∗^	0.198

^∗^ *P* < 0.005 vs. predialysis, and data is presented as mean with SD.

## Data Availability

The Excel files of individual clinical data used to support the findings of this study are available from the corresponding author upon request.
